# An Accurate and Effective Method for Measuring Osimertinib by UPLC-TOF-MS and Its Pharmacokinetic Study in Rats

**DOI:** 10.3390/molecules23112894

**Published:** 2018-11-06

**Authors:** Song-Tao Dong, Ying Li, Hao-Tian Yang, Yin Wu, Ya-Jing Li, Cong-Yang Ding, Lu Meng, Zhan-Jun Dong, Yuan Zhang

**Affiliations:** 1 National Clinical Drug Monitoring Center, Department of Pharmacy, Hebei Province General Center, Shijiazhuang 050051, China; dongsongtao8886@163.com (S.-T.D.); lyyaoda@126.com (Y.L.); yanghaotian0917@163.com (H.-T.Y.); Wuyin82@126.com (Y.W.); 15369305382@163.com (Y.-J.L.); dingcy1989@126.com (C.-Y.D.); M18203213683@163.com (L.M.); dzjhbgh@126.com (Z.-J.D.); 2 Department of Pharmaceutics, School of Pharmacy, China Medical University, Shenyang 110001, China; 3 Department of Pharmacy, National Cancer Center/National Clinical Research Center for Cancer, Chinese Academy of Medical Sciences and Peking Union Medical College, Beijing 100021, China

**Keywords:** osimertinib, UPLC-TOF-MS, rat, pharmacokinetics

## Abstract

Osimertinib, a new-generation inhibitor of the epidermal growth factor, has been used for the clinical treatment of advanced T790M mutation-positive tumors. In this research, an original analysis method was established for the quantification of osimertinib by ultra-performance liquid chromatography with time of flight mass spectrometry (UPLC-TOF-MS) in rat plasma. After protein precipitation with acetonitrile and sorafinib (internal standard, IS), they were chromatographed through a Waters XTerra MS C_18_ column. The mobile phase was acetonitrile and water (including 0.1% ammonia). The relative standard deviation (RSD) of the intra- and inter-day results ranged from 5.38 to 9.76% and from 6.02 to 9.46%, respectively, and the extraction recovery and matrix effects were calculated to range from 84.31 to 96.14% and from 91.46 to 97.18%, respectively. The results illustrated that the analysis method had sufficient specificity, accuracy and precision. Meanwhile, the UPLC-TOF-MS method for osimertinib was successfully applied into the pharmacokinetics of SD rats.

## 1. Introduction

**Figure 1:**
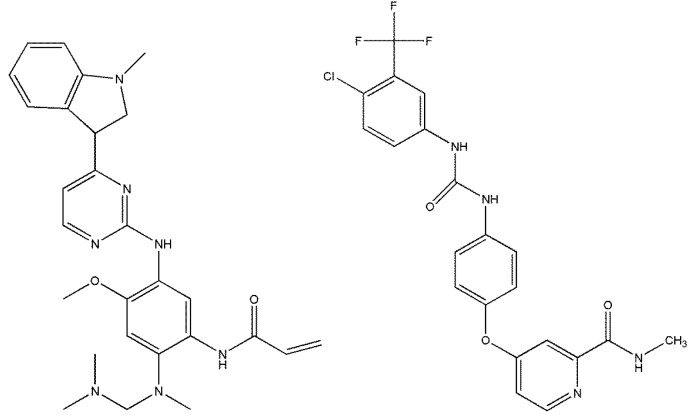
Structures of osimertinib (molecular weight = 499.619 Da) and sorafenib (molecular weight = 464.825 Da).

Osimertinib (AZD9291, Merelitinib, Tagriiso^©^), *N*-(2-{2dimethylaminoethyl-methylamino}-4-methoxy-5-{[4-(1-methylindol-3-yl)pyrimidin-2yl]amino}phenyl)prop-2-enamide (Figure 1), a third-generation, highly selective, irreversible covalent inhibitor has been created by AstraZeneca for the clinical therapy of advanced non-small cell lung cancer (NSCLC) [1,2,3,4,5,6]. NSCLC patients, who have epidermal growth factor receptor (EGFR) tyrosine kinase inhibitor (TKI) resistance, are mostly subject to a mutation of EGFR. Therefore, this has been the active target for the osimertinib [7,8,9]. In addition, the tablet formulation of osimertinib has been approved by the FDA (Food and Drug Administration of the USA) for NSCLC patients, who have progressed to or completed EGFR TKI therapy in 2015.

To the best of our knowledge, several papers have established the methods for the determination of osimertinib in biological samples, and the utilized apparatuses are all ultra-performance liquid chromatography coupled with tandem mass spectrometry (UPLC-MS/MS) [10,11,12,13]. There are some advantages of UPLC-MS/MS, including its high sensitivity, high stability, and short analytic time. Unlike UPLC-MS/MS, UPLC-TOF-MS has specific advantages such as its high working efficiency, wide measurable mass range, and high ratio of resolution [14,15,16,17]. In addition, the capability of simultaneous quantitative analysis and qualitative analysis greatly benefits the analysts, and it is very useful for the further study of agents, such as their metabolism, enzymology, transportation and so on [18]. However, to date, methods using UPLC-TOF-MS for the determination of osimertinib have not been reported. In the present study, we are the first to quantify osimertinib in rat plasma using UPLC-TOF-MS.

The objective of this study was to investigate a specific, sensitive, rapid and reliable UPLC-TOF-MS method for osimertinib quantification in rat plasma samples. Meanwhile, we have successfully investigated the pharmacokinetic study of osimertinib in rats using this UPLC-TOF-MS method.

## 2. Results and Discussion

### 2.1. UPLC-TOF-MS Method Development

**Figure 2:**
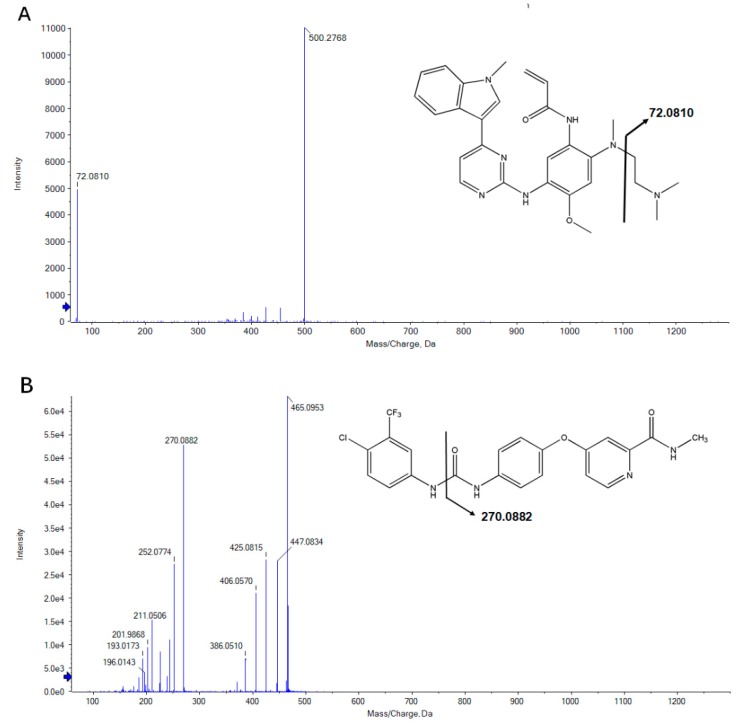
Product ion scan of osimertinib 500.2768 → 72.0810 (**A**), and IS 465.0953 → 270.0882 (**B**).

By using the product scan mode, we could find the method of pyrolysis of osimertinib and ion of sorafinib (IS) in rat plasma under the UPLC-TOF-MS condition that we have optimized (see Section 2.3) (Figure 2). The parent ion m/z of osimertinib was 500.2768 and the characteristic product ion was 72.0810. In addition, the m/z of the parent ion and product ion of sorafenib (IS) were 465.0953 and 270.0882, respectively. All these results are consistent with previous studies [4,5,6,7,19].

**Figure 3:**
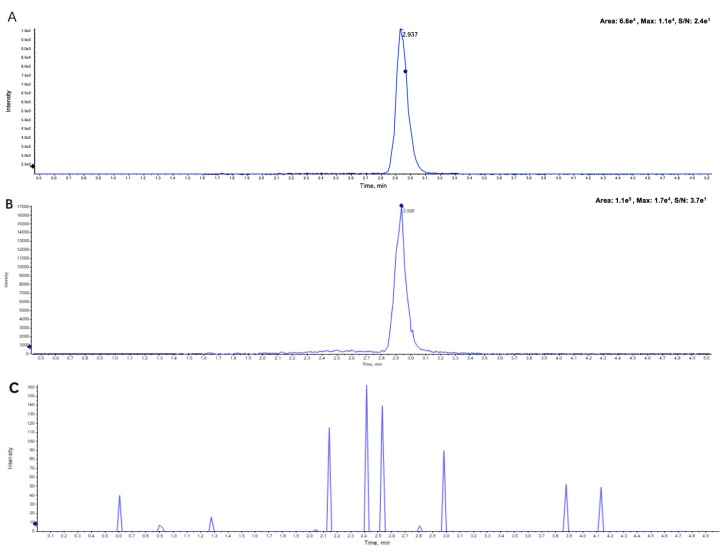

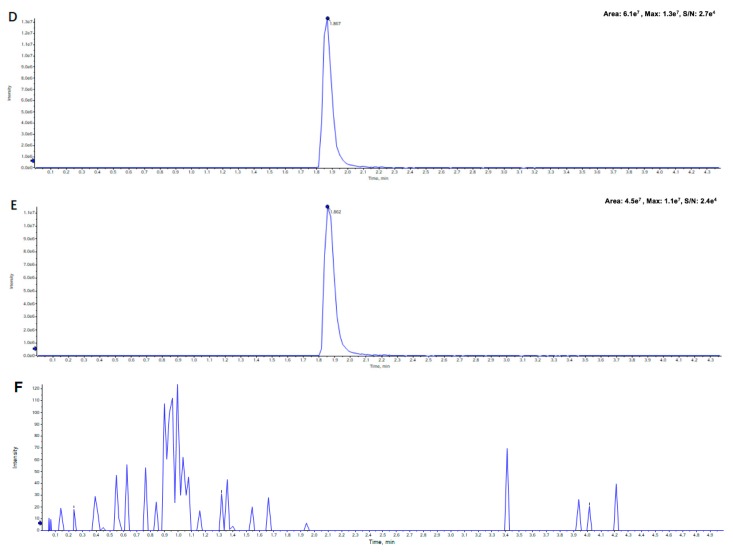
Typical chromatograms of (**A**) standard osimertinib (20 ng/mL) in rat plasma, (**B**) pharmacokinetic plasma sample, (**C**) blank plasma, (**D**) standard ion of sorafenib (IS) (500 ng/mL) in rat plasma, (**E**) pharmacokinetic plasma sample, and (**F**) blank plasma.

However, the optimization of the UPLC condition became the key point in the process of investigating our UPLC-TOF-MS method. We used various proportions and gradients of water (containing 0.1% formic acid)–acetonitrile and water (containing 0.1% formic-ammonia formate)–acetonitrile to optimize the chromatographic conditions. However, the chromatographic peaks all displayed the trailing phenomenon (Figure S1). Since the pKa of osimertinib was 13.64, it needs a basic environment to keep its molecular state. Therefore, we decided to use 0.1% ammonia water–acetonitrile as the mobile phase, which enabled us to finally obtain the symmetrical chromatographic peak (Figure 3).

### 2.2. Method Validation

#### 2.2.1. Specificity and Selectivity

The typical chromatograms of blank plasma, the plasma sample spiked with osimertinib and IS and rat plasma after treatment are shown in Figure 3. Figure 3A–C displays the characteristic peaks of standard osimertinib in rat plasma, the pharmacokinetic plasma sample and blank plasma, and the chromatograms showed a good specificity. In addition, Figure 3D–F exhibits the good specificity of standard sorafenib (IS). Moreover, these results also showed that there was no significant chromatographic interference with the osimertinib and IS in rat plasma.

#### 2.2.2. Calibration and Lower Limit of Quantification (LLOQ)

To investigate the linearity of osimertinib and IS, nine calibration concentrations were analyzed in each validation batch, and the results showed the good linearity (*r*^2^ > 0.99) of the calibration curve of osimertinib and IS over the range of 1 to 500 ng/mL. The lower limits of quantification (LLOQ) of osimertinib and IS were both 1 ng/mL, and the ratios of signal-to-noise were considerably higher than 5. In addition, the LLOQ of this UPLC-TOF-MS method was sufficient for the determination of the osimertinib pharmacokinetic study.

#### 2.2.3. Precision and Accuracy

The intra- and inter-day precision and accuracy are shown in Table 1. In addition, the intra- and inter-day results of the HQC, MQC and LQC are investigated with the RSD ranging from 5.38–9.76% (intra-day) and 6.02–9.46% (inter-day), respectively. The results ranged within the standard acceptance limit of 15% and demonstrated the good accuracy and precision of osimertinib.

#### 2.2.4. Extraction Recovery and Matrix Effect

Table 2 shows the extraction recovery of osimertinib and IS, and these results are sufficient for quantification. The matrix effects of osimertinib and IS range from 0.810 to 0.926 and from 0.798 to 0.934, respectively (Table 3). The calibration curves of the final concentrations of IS were the same as those of osimertinib. The results showed the high extraction recovery and lack of significant matrix effect of this method for osimertinib and IS in the rat plasma.

#### 2.2.5. Stability

Table 4 shows the stability of HQC, MQC and LQC of osimertinib under four different storage conditions. According to the results, osimertinib was found to have good stability at room temperature (25 °C) and the autosampler temperature (4 °C) for 24 h and remained stable following three freeze (−80 °C) and thaw (0 °C) cycles. Moreover, the plasma samples of osimertinib were also stable at the storage temperature (−80 °C) for at least 30 days.

### 2.3. Pharmacokinetic Application

**Figure 4:**
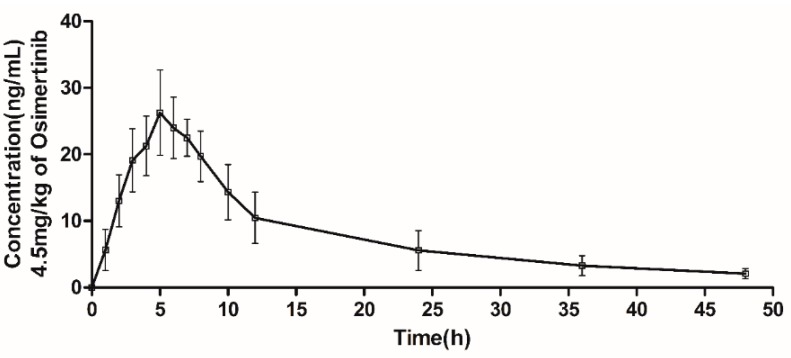
Plasma concentration-time profile after single oral administration of osimertinib (4.5 mg/kg) to rats. Data are expressed as the mean ± SD (*n* = 7).

After successfully establishing the analysis method of osimertinib by UPLC-TOF-MS, we applied this method into the pharmacokinetic study in SD rats. The dosage of the oral administration was 4.5 mg/kg and the mean plasma time-concentration of osimertinib in seven rats is shown in Figure 4. This showed that the osimertinib had the highest plasma concentration at 4.5 h after oral administration and the C_max_ was 28.49 ng/mL. In addition, Table 5 shows the following non-compartmental parameters of osimertinib in SD rats: a terminal half-life of (14.96 ± 3.44) h, a distribution volume of (233.82 ± 66.68) L/kg, and a clearance of about (10.84 ± 1.94) L/h/kg (Table 5). These pharmacokinetic data of osimertinib can provide more information regarding its application in clinical treatment.

## 3. Materials and Methods

### 3.1. Drugs and Materials

Osimertinib (purity >99%) and sorafenib (internal standard, IS) were purchased from Stanford Analytical Chemicals Inc. (Eugene, OR, USA). Dimethyl sulfoxide (DMSO) was purchased from Beijing Solarbio Science and Technology Co., Ltd. (Beijing, China). Ammonia and acetonitrile (HPLC grade) were provided by Tianjin Kemiou Chemical Reagent Co., Ltd. (Tianjin, China) and the Fisher Scientific Co., Ltd. (Pittsburgh, PA, USA), respectively. Ultrapure water was obtained from a milli-Q reagent water purification system (Millipore, Bedford, MA, USA).

### 3.2. Apparatus

The UPLC-TOF-MS/MS method was performed on a system that includes a Qtrap 5600-TOF mass spectrometer (AB Sciex, MA, USA) and an UPLC chromatographic analysis system (Shimadzu, Kyoto, Japan). An Xterra MS C_18_ column (100 × 2.1 mm, 3.5 μm) (Waters Corp., Milford, MA, USA) was used for the analytical separation at the temperature of 40 °C. A TGL-16M high speed centrifuge was purchased from Cence Co., Ltd. (Changsha, China).

### 3.3. Solution Preparation

DMSO was used to dissolve the accurately weighed standard of osimertinib and sorafenib (IS) to obtain the stock solutions of 1.0 mg/mL. Then, the working solutions of osimertinib were diluted serially with 50% acetonitrile in water to achieve 10, 20, 50, 100, 200, 500, 1000, 2000 and 5000 ng/mL. Next, 10 μL diluted solutions were diluted in 100 μL blank plasma to obtain the final calibration standard samples, and the range of the final concentrations of the calibration standards was from 1 to 500 ng/mL.

The working solution concentration of IS was 500 ng/mL, which was dissolved using 50% acetonitrile water (*v*/*v*). The quality controls (QCs) were diluted to achieve the lower limit of the quantification (10 ng/mL, LLOQ), low (20 ng/mL, LQC), medium (200 ng/mL, MQC), and high (4000 ng/mL, HQC) concentrations of osimertinib. After that step, 10 μL of the QC solutions were dissolved by 100 μL of blank plasma to get the final concentrations of LLOQ (1 ng/mL), LQC (2 ng/mL), MQC (20 ng/mL), HQC (400 ng/mL) and 50 ng/mL of IS. All samples and working solutions were kept at −20 °C before use.

### 3.4. UPLC-TOF-MS Condition

The chromatographic separation was performed using a C_18_ column, and its temperature was kept at 40 °C. The chromatographic separation consisted of a 0.1% ammonia (A) and acetonitrile (B) mixture and a 0.4 mL/min flow rate was maintained. The gradient ran linearly from 10% to 95% between 0 and 1.5 min, and then the mobile phase was kept at 95% for 5.0 min. 3.0 μL of samples was injected into the analysis system and the total analytical time of one sample was 5.1 min. The temperature of the autosampler was maintained at 4 °C.

The TOF-MS spectrometer was set up in the positive ion full scan electrospray and high sensitivity mode with an m/z range from 100 to 1000 Da, and the accumulation time was set as 0.25s. The parameters of TOF-MS were as follows: nebulizer gas (gas 1), 55 psi; heater gas (gas 2), 55 psi; curtain gas, 35 psi; ion spray voltage, 5500 V; turbo spray temperature, 550 °C; declustering potential (DP), 100 V; and collision energy (CE), 35 eV. The conditions of the information-dependent data acquisition (IDA) criteria were as follows. The eight most intense fragment ions of each analyte in 100 cps were chosen as the product ions, and the m/z of the product ions ranged from 600 to 1300 over a 0.08 s accumulation time. In addition, the CE and collision energy scope (CES) of the product ions scan were set at 35 eV and 15 eV, respectively.

### 3.5. Pharmacokinetic Application

The pharmacokinetic study of osimertinib was applied to seven male SD rats using oral administration by gavage and the dosage was 4.5 mg/kg. Osimertinib was diluted and suspended by 0.5% sodium carboxymethylcellulose. No less than 0.3 mL rat plasma was sampled at 0, 1, 2, 3, 4, 5, 6, 7, 8, 10, 12, 24, 36, and 48 h after oral administration through the oculi chorioideae vein under the condition of light ether anesthesia. After 10 min centrifuging of all analytes at 5000× *g*, the supernatant was collected and frozen at −40 °C until analysis. The use of animals in the presented study was permitted by the Ethics Committee of the Hebei Medical University, and all animal studies were carried out according to the Guidance for the Care and Use of Laboratory Animals of the US National Institute of Health.

### 3.6. Sample Preparation

Ten microliters of IS solution (500 ng/mL) was prepared in 100 μL rat plasma and vortex-mixed for 20 s. After that step, 500 μL of acetonitrile was added to precipitate the protein. Then, the mixture was vortexed for 1 min and centrifuged at 12,000 rpm for 10 min. The supernatant was transferred into a new Eppendorf tube and evaporated to dryness through nitrogen gas at 45 °C. After 100 μL of acetonitrile was added to reconstitute the residue and vortexed for 1 min, all samples were centrifuged at 12,000× *g* for 10 min. Finally, 80 μL of the supernatant was collected and injected into UPLC sample vials before use.

### 3.7. Method Validation

According to the US Food and Drug Administration (FDA) guidelines regarding the bioanalytical method’s validation [20], the method validation was investigated and established, including the specificity and selectivity, the linearity and sensitivity, the recovery, the stability, and the precision, accuracy and matrix effect.

#### 3.7.1. Selectivity and Specificity

The selectivity and specificity of the developed method was assessed by comparing the chromatography of the blank plasma and blank plasma spiked with the targets.

#### 3.7.2. Linearity and Sensitivity

A series of calibration analytes from 1 to 500 ng/mL consisted of the calibration curve of osimertinib. In addition, the method to determine the linearity of calibration curve was the peak area ratios of osimertinib and IS, and these ratios were used to get a least-squares weighted regression (the weighting factor was 1/y, and y = peak area ratio of osimertinib/IS). The correlation coefficient (*r*^2^) of all calibration curves, which were desirable for this method, were better than or equal to 0.99.

LLOQ was used to investigate the method sensitivity and follows these two criteria: (1) the comparison of the LLOQ and blank response should occur at least 5 times; and (2) the analyte peak of the LLOQ should be discrete, reproducible and identifiable, and its accuracy and precision should be at least 20%.

#### 3.7.3. Precision, Accuracy and Matrix Effect

The intra-day accuracy and precision were investigated through the determination of six QC analytes of the high (HQC = 400 ng/mL), medium (MQC = 20 ng/mL), low (LQC = 2 ng/mL), and LLOQ (LLOQ = 1 ng/mL) concentrations. The inter-day accuracy and precision were conducted through the determination of the six replicates of the four levels of QCs using the same preparation on 3 separate days. The assay accuracy of the QC samples was compared to the corresponding standard calibration concentration. The precision of the replicates was evaluated by the RSD (relative standard deviation).

It can be accepted that the mean values of accuracy should not exceed 15% at the HQC and MQC, and LQC concentrations and LLOQ should not exceed 20%. Similarly, the relative standard deviation of the precision for HQC, MQC, and LQC concentration levels should not exceed 15% and the limitation of LLOQ was 20%.

The matrix effect was determined by dividing slopes of calibration curves of osimertinib in the rat blood matrix and mobile phase.

#### 3.7.4. Recovery

The extraction recovery of osimertinib was evaluated by comparing the peak area ratios of standard solution samples and the same concentrations rat plasma samples through five replicates at HQC, MQC and LQC concentrations.

#### 3.7.5. Stability

The stabilities of this method, which include the freeze–thaw stability, autosampler stability, short-term stability and long-term stability, were evaluated through three QC samples after the sample preparation method. The freeze (−80 °C)–thaw (room temperature) stability was conducted under the conditions of three free–thaw cycles. The autosampler stability of the plasma samples was investigated by the extracted QC samples that were kept in an autosampler (4 °C) for 12 h. In addition, the long-term stability was evaluated through the determination of three QC samples that were kept at −80 °C for 30 days. All samples were considered stable with RSDs < ±15%.

### 3.8. Data Analysis

Dynamic background subtraction is a novel technique performed using the Analyst software (AB Sciex, Foster City, CA, USA). In addition, the data of the pharmacokinetic study of osimertinib were collected and calculated by the DAS 2.1.1 software in the non-compartmental mode (Mathematical Pharmacology Professional Committee of China, Shanghai, China).

## 4. Conclusions

A sensitive UPLC-TOF-MS method for the determination of osimertinib has been established in this research. The method exhibited excellent precision, recovery and sensitivity. The results indicated that UPLC-TOF-MS could serve as a highly interesting analytical alternative for bioanalysis.

## Figures and Tables

**Table 1:** Intra- and inter-day precision and accuracy of osimertinib in rat plasma. RSD: relative standard deviation.

Concentration (ng/mL)	Intra-Day (n = 7)			Inter-Day (n = 7)		
	Measured Conc. (ng/mL)	Precision, RSD (%)	Accuracy (%)	Measured Conc. (ng/mL)	Precision RSD (%)	Accuracy (%)
400	395.12 ± 21.27	5.38	98.78	401.13 ± 24.15	6.02	100.28
20	21.08 ± 1.42	6.74	105.40	19.34 ± 1.83	9.46	96.70
2	2.05 ± 0.20	9.76	102.50	1.98 ± 0.17	8.59	99.00

**Table 2:** Extraction recovery and matrix effect of osimertinib and IS in rat plasma.

Analyte	Concentration (ng/mL)	Extraction Recovery (%)	
		Mean ± SD	RSD
Osimertinib	400	95.24 ± 3.01	3.16
	20	96.14 ± 1.83	1.90
	2	84.31 ± 3.18	3.77
Sorafinib	500	87.22 ± 4.23	4.85

**Table 3:** The slope ratio of the solvent linear equation and the matrix linear equation.

Analyte	Calibration Curve	R^2^	R_slope_		
			Min	Max	ΔR
Osimertinib	Y = 0.085X + 0.1102	0.9997	0.810	0.926	0.116
Sorafinib	Y= 0.0781X + 0.2314	0.9996	0.798	0.934	0.136

R_slope_ = Slope of matrix standard calibration curve/slope of mobile phase standard calibration curve.

**Table 4:** Stability of osimertinib in rat plasma under various storage conditions.

Storage Condition	Concentration (ng/L)	Mean ± SD	RSD%
Autosampler (4 °C) temperature for 24 h	2	2.12 ± 0.23	10.85
	20	21.45 ± 1.81	8.44
	400	406.81 ± 5.64	1.39
Room temperature (25 °C) for 24 h	2	2.21 ± 0.26	3.66
	20	22.45 ± 2.18	9.71
	400	407.28 ± 5.12	1.26
Storage temperature (−80 °C) for 30 days	2	2.29 ± 0.25	10.92
	20	21.33 ± 1.74	8.16
	400	406.34 ± 7.51	1.85
Three freeze–thaw cycles (each at −80 °C for 24 h)	2	2.27 ± 0.19	8.37
	20	22.20 ± 1.92	8.65
	400	406.17 ± 6.19	1.52

**Table 5:** Pharmacokinetic parameters of osimertinib after oral administration of 4.5 mg/kg to rats.

Pharmacokinetic Parameter	Osimertinib
*AUC*_(0–t)_, ng/mL·h	382.00 ± 69.00
*AUC*_(0–∞)_, ng/mL·h	426.01 ± 81.73
MRT, h	14.51 ± 1.91
t_1/2z_, h	14.96 ± 3.44
t_max_, h	4.80 ± 1.10
C_max_, ng/mL	28.49 ± 3.97
V_z_/F, L/kg	233.82 ± 66.68
CL_z_/F, L/h/kg	10.84 ± 1.94

Data are expressed as the mean ± SD (*n* = 7).

## Supplementary Materials

The following are available online.

## References

[1] Chen Z., Chen Y., Xu M., Chen L., Zhang X., To K.K., Zhao H., Wang F., Xia Z., Chen X. (2016). Osimertinib (AZD9291) enhanced the efficacy of chemotherapeutic agents in ABCB1- and ABCG2-overexpressing cells in vitro, in vivo, and ex vivo. Mol. Cancer Ther..

[2] Cross D.A., Ashton S.E., Ghiorghiu S., Eberlein C., Nebhan C.A., Spitzler P.J., Orme J.P., Finlay M.R., Ward R.A., Mellor M.J. (2014). AZD9291, an irreversible EGFR TKI, overcomes T790M-mediated resistance to EGFR inhibitors in lung cancer. Cancer Discov..

[3] Yang M., Tong X., Xu X., Zheng E., Ni J., Li J., Yan J., Shao Y.W., Zhao G. (2018). Case Report: Osimertinib achieved remarkable and sustained disease control in an advanced non-small-cell lung cancer harboring EGFR H773L/V774M mutation complex. Lung Cancer.

[4] Gao X., Le X., Costa D.B. (2016). The safety and efficacy of osimertinib for the treatment of EGFR T790M mutation positive non-small-cell lung cancer. Expert Rev. Anticancer Ther..

[5] Ricciuti B., Chiari R., Chiarini P., Crino L., Maiettini D., Ludovini V., Metro G. (2016). Osimertinib (AZD9291) and CNS response in two radiotherapy-naive patients with EGFR-Mutant and T790M-Positive advanced non-small cell lung cancer. Clin. Drug Investing..

[6] Uchino J., Nakao A., Tamiya N., Kaneko Y., Yamada T., Yoshimura K., Fujita M., Takayama K. (2018). Treatment rationale and design of the SPIRAL study: A phase II trial of osimertinib in elderly epidermal growth factor receptor T790M-positive nonsmall-cell lung cancer patients who progressed during prior EGFR-TKI treatment. Medicine.

[7] Masuhiro K., Shiroyama T., Suzuki H., Takata S.O., Nasu S., Takada H., Morita S., Tanaka A., Morishita N., Okamoto N. (2018). Impact of pleural effusion on outcomes of patients receiving Osimertinib for NSCLC harboring EGFR T790M. Anticancer Res..

[8] Mountzios G. (2018). Making progress in epidermal growth factor receptor (EGFR)-mutant non-small cell lung cancer by surpassing resistance: Third-generation EGFR tyrosine kinase inhibitors (EGFR-TKIs). Ann. Transl. Med..

[9] Aguiar P.N., Haaland B., Park W., San Tan P., Del Giglio A., de Lima Lopes G. (2018). Cost-effectiveness of Osimertinib in the First-Line treatment of patients with EGFR-Mutated advanced non-small cell lung cancer. JAMA Oncol..

[10] Rood J.J.M., van Bussel M.T.J., Schellens J.H.M., Beijnen J.H., Sparidans R.W. (2016). Liquid chromatography-tandem mass spectrometric assay for the T790M mutant EGFR inhibitor osimertinib (AZD9291) in human plasma. J. Chromatogr. B Analyt. Technol. Biomed. Life Sci..

[11] Xiong S., Deng Z., Sun P., Mu Y., Xue M. (2017). Development and Validation of a Rapid and Sensitive LC-MS/MS Method for the Pharmacokinetic Study of Osimertinib in Rats. J. AOAC Int..

[12] Reis R., Labat L., Allard M., Boudou-Rouquette P., Chapron J., Bellesoeur A., Thomas-Schoemann A., Arrondeau J., Giraud F., Alexandre J. (2018). Liquid chromatography-tandem mass spectrometric assay for therapeutic drug monitoring of the EGFR inhibitors afatinib, erlotinib and osimertinib, the ALK inhibitor crizotinib and the VEGFR inhibitor nintedanib in human plasma from non-small cell lung cancer patients. J. Pharm. Biomed. Anal..

[13] Zheng X., Wang W., Zhang Y., Ma Y., Zhao H., Hu P., Jiang J. (2018). Development and validation of a UPLC-MS/MS method for quantification of osimertinib (AZD9291) and its metabolite AZ5104 in human plasma. Biomed. Chromatogr..

[14] Lee M.J., Park J.S., Choi D.S., Jung M.Y. (2013). Characterization and quantitation of anthocyanins in purple-fleshed sweet potatoes cultivated in Korea by HPLC-DAD and HPLC-ESI-QTOF-MS/MS. J. Agric. Food Chem..

[15] Park J.S., Jung M.Y. (2012). Development of high-performance liquid chromatography-time-of-flight mass spectrometry for the simultaneous characterization and quantitative analysis of gingerol-related compounds in ginger products. J. Agric. Food Chem..

[16] Shintani-Ishida K., Kakiuchi Y., Ikegaya H. (2016). Successful quantification of 4’-methyl-alpha-pyrrolidinohexanophenone (MPHP) in human urine using LC-TOF-MS in an autopsy case. Forensic Toxicol..

[17] Wille K., Kiebooms J.A., Claessens M., Rappe K., Vanden Bussche J., Noppe H., Van Praet N., De Wulf E., Van Caeter P., Janssen C.R. (2011). Development of analytical strategies using U-HPLC-MS/MS and LC-ToF-MS for the quantification of micropollutants in marine organisms. Anal. Bioanal. Chem..

[18] Zhang H., Henion J. (2001). Comparison between liquid chromatography-mass spectrometry for quantitative determination of idocifene in human plasma. J. Chromatogr. B Biomed. Sci. Appl..

[19] Abdelhameed A.S., Attwa M.W., Kadi A.A. (2017). An LC-MS/MS method for ripad and sensitive high-throughput simultaneous determination of various protein kinase inhibitors in human plasma. Biomed. Chromatogr..

[20] Smeraglia J., McDougall S., Elsby K., Companjen A., White S., Golob M., Brudny-Kloeppel M., Amsterdam P., Timmerman P. (2014). Conference report: AAPS and US FDA Crystal City V meeting on Quantitative Bioanalytical Method Validation and Implementation: Feedback from the EBF. Bioanalysis.

