# Supplementary material for: An Accurate and Effective Method for Measuring Osimertinib by UPLC-TOF-MS and Its Pharmacokinetic Study in Rats
Source: Molecules. 2018 Nov 6;23(11):2894. doi: 10.3390/molecules23112894 (PMC6278556; doi:10.3390/molecules23112894)
Supplement: Supplementary file 1 [file molecules-23-02894-s001.zip › Supplyment/Supplyment 2.docx]

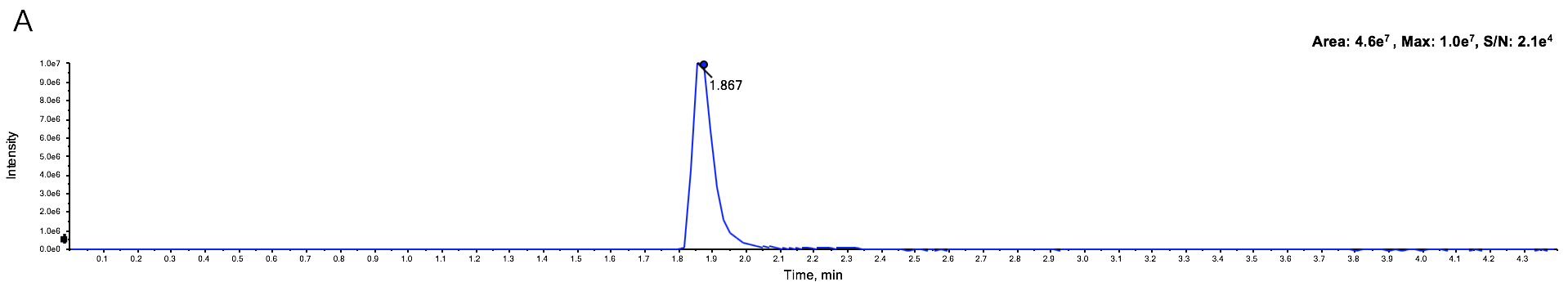


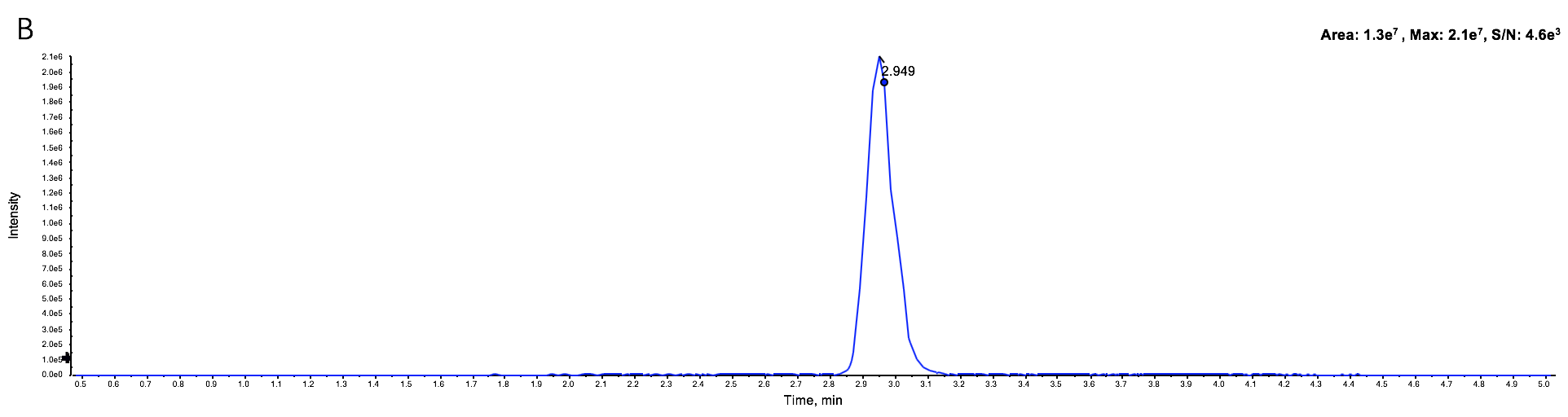


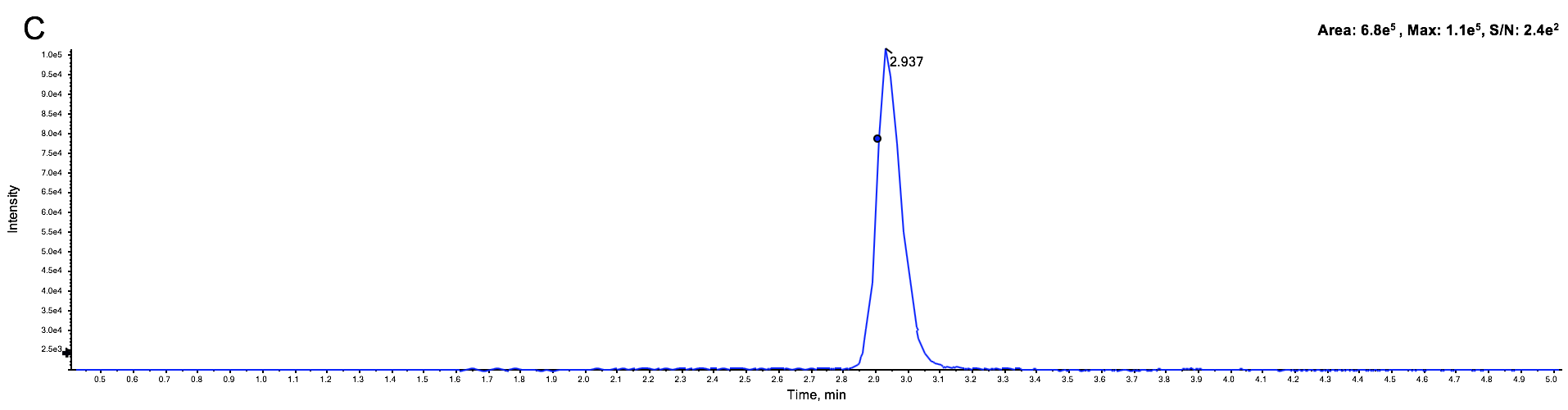


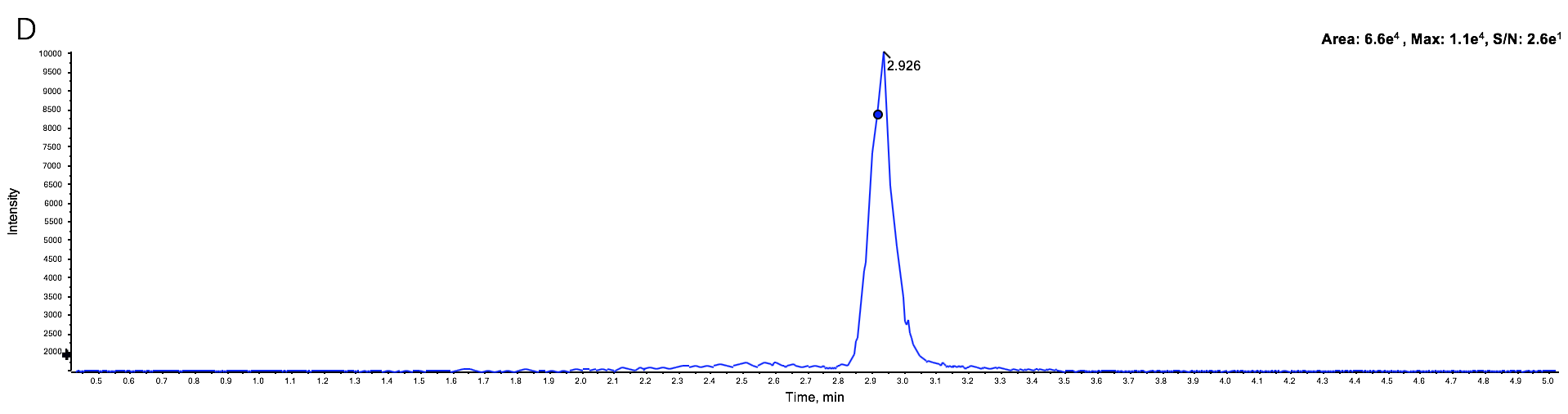


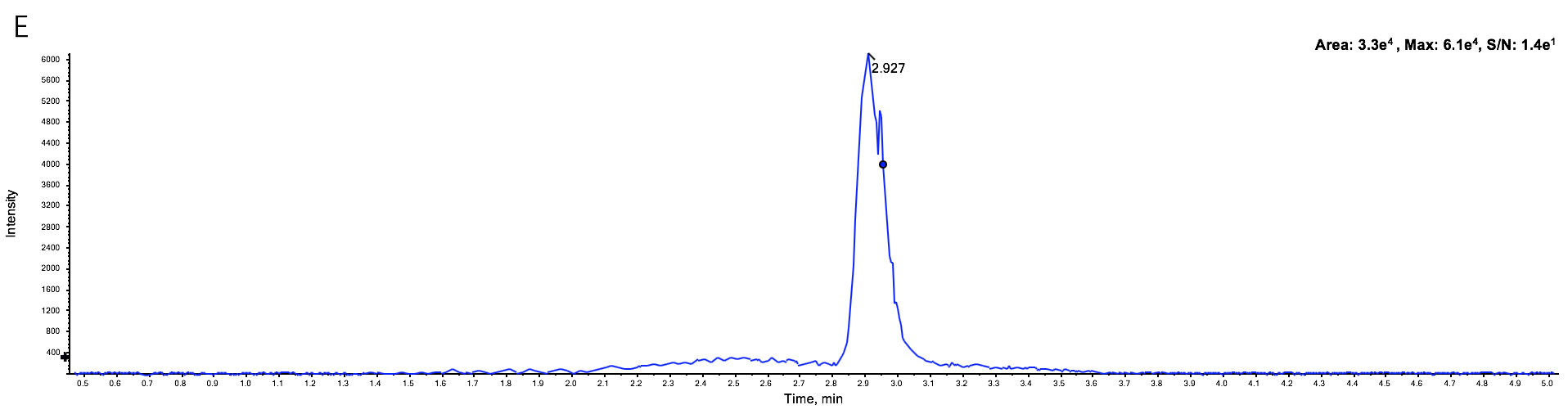


**Supplementary table.** The area, height and S/N of sorafenib and osimertinib.

| Analyte | Concentration (ng/mL) | Number | Area | Height | S/N |
| --- | --- | --- | --- | --- | --- |
| Sorafenib | 500 | A | 4.6e^7^ | 1.0e^7^ | 2.1e^4^ |
| Osimertinib | 400 | B | 1.3e^7^ | 2.1e^6^ | 4.6e^3^ |
|  | 20 | C | 6.8e^5^ | 1.1e^5^ | 2.4e^2^ |
|  | 2 | D | 6.6e^4^ | 1.1e^4^ | 2.6e^1^ |
|  | 1 | E | 3.3e^4^ | 6.1e^3^ | 1.4e^1^ |
