# Supplementary material for: An Accurate and Effective Method for Measuring Osimertinib by UPLC-TOF-MS and Its Pharmacokinetic Study in Rats
Source: Molecules. 2018 Nov 6;23(11):2894. doi: 10.3390/molecules23112894 (PMC6278556; doi:10.3390/molecules23112894)
Supplement: Supplementary file 1 [file molecules-23-02894-s001.zip › Supplyment/Supplyment 2.pdf]

A Area: $4.6e^{7}$ Height: $1.0e^{7}$

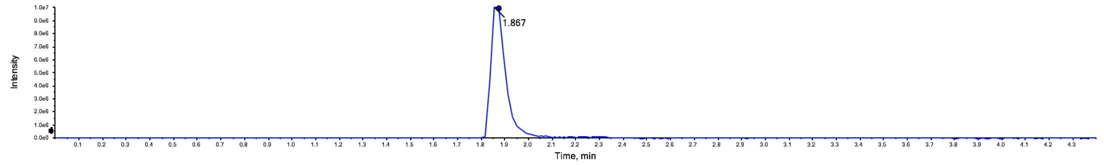

B Area: $1.3e^{6}$ Height: $2.1e^{5}$

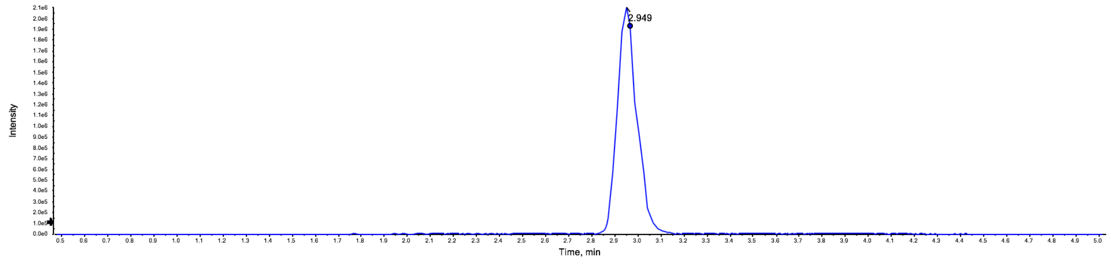

C Area: $6.8e^{5}$ Height: $1.1e^{5}$

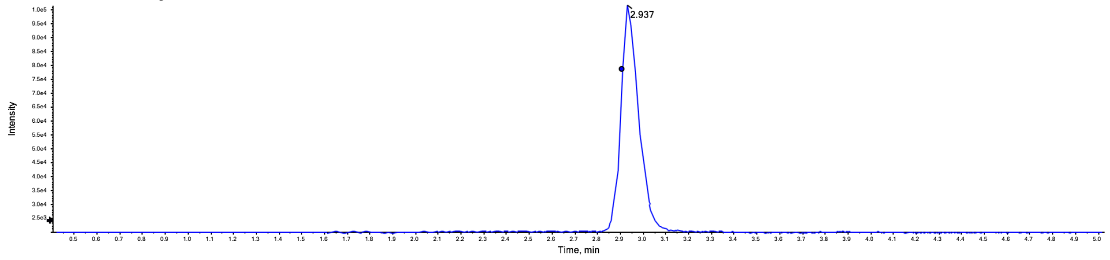

D Area: $6.6e^{4}$ Height: $1.1e^{4}$

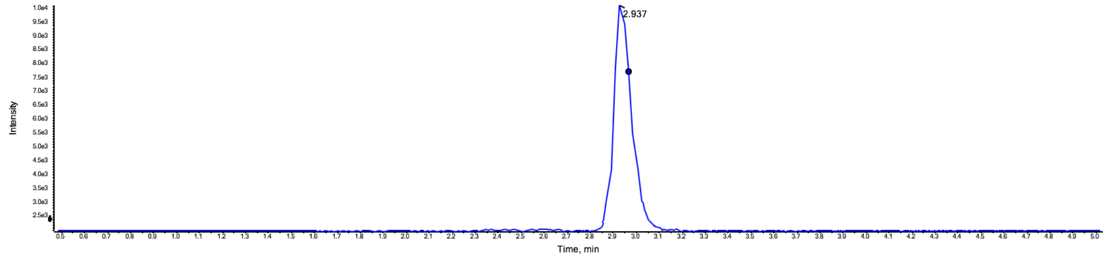

E Area: $3.3e^{4}$ Height: $6.1e^{3}$

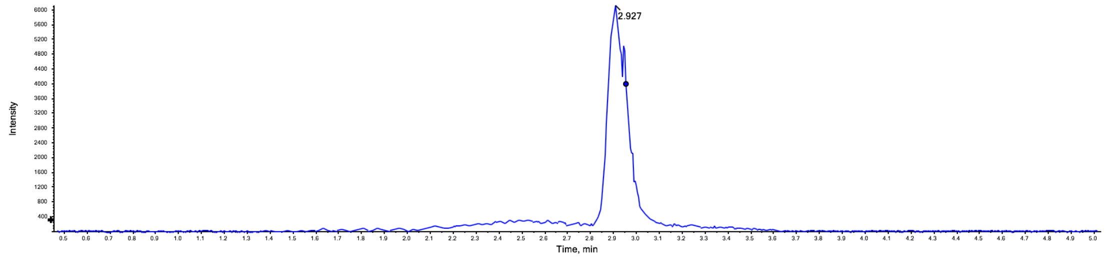

Number	Concentration (ng/mL)	Area	Height
A	500	$4.6e^{7}$	$1.0e^{7}$
B	400	1.3e7	2.1e6
C	20	6.8e5	1.1e5

D	2	6.6e4	1.1e4
E	1	3.3e4	6.1e3
