# Supplementary material for: An Accurate and Effective Method for Measuring Osimertinib by UPLC-TOF-MS and Its Pharmacokinetic Study in Rats
Source: Molecules. 2018 Nov 6;23(11):2894. doi: 10.3390/molecules23112894 (PMC6278556; doi:10.3390/molecules23112894)
Supplement: Supplementary file 1 [file molecules-23-02894-s001.zip › Supplyment/Supplement 1.pdf]

Supplement 1

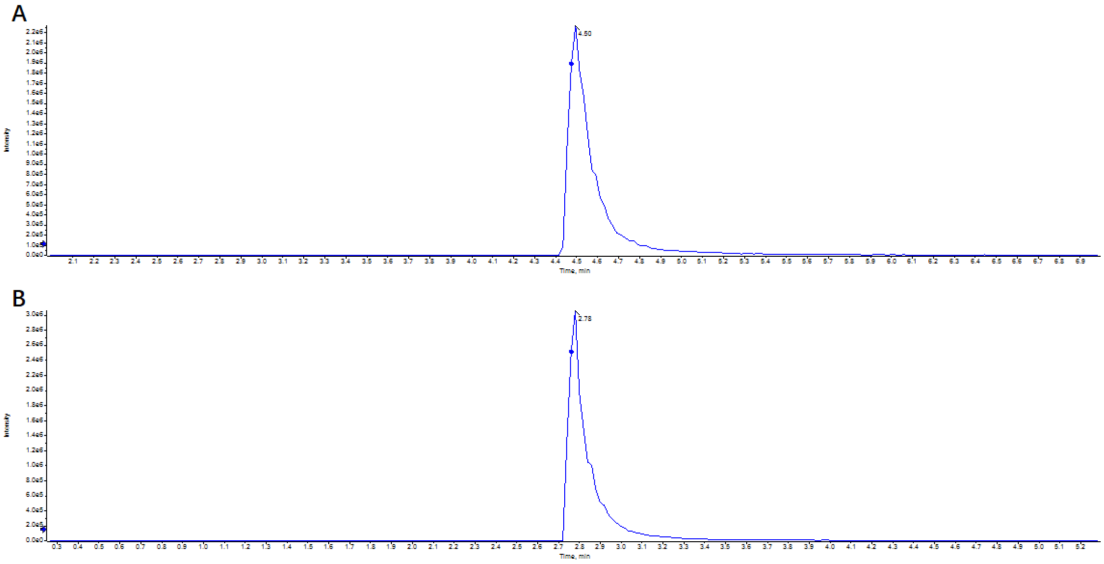

(A) Chromatographic peak of osimertinib under 0.1% formic acid water and acetonitrile, and (B) Chromatographic peak of osimertinib under 0.1% formic acid-ammonia formate water and acetonitrile.
